# Engineered Tissue Inhibitor of Metalloproteinases-3 Variants Resistant to Endocytosis Have Prolonged Chondroprotective Activity*

**DOI:** 10.1074/jbc.M116.733261

**Published:** 2016-08-31

**Authors:** Christine M. Doherty, Robert Visse, Deendayal Dinakarpandian, Dudley K. Strickland, Hideaki Nagase, Linda Troeberg

**Affiliations:** From the ‡ Arthritis Research UK Centre for Osteoarthritis Pathogenesis, Kennedy Institute of Rheumatology, Nuffield Department of Orthopaedics, Rheumatology, and Musculoskeletal Sciences, University of Oxford, Roosevelt Drive, Oxford OX3 7FY, United Kingdom,; the § School of Computing and Engineering, University of Missouri, Kansas City, Missouri 64111, and; the ¶ University of Maryland School of Medicine, Baltimore, Maryland 21201

**Keywords:** ADAM, ADAMTS, extracellular matrix, glycosaminoglycan, osteoarthritis, receptor, tissue inhibitor of metalloproteinase (TIMP), low-density lipoprotein receptor-related protein 1

## Abstract

Tissue inhibitor of metalloproteinases-3 (TIMP-3) is a central inhibitor of matrix-degrading and sheddase families of metalloproteinases. Extracellular levels of the inhibitor are regulated by the balance between its retention on the extracellular matrix and its endocytic clearance by the scavenger receptor low density lipoprotein receptor-related protein 1 (LRP1). Here, we used molecular modeling to predict TIMP-3 residues potentially involved in binding to LRP1 based on the proposed LRP1 binding motif of 2 lysine residues separated by about 21 Å and mutated the candidate lysine residues to alanine individually and in pairs. Of the 22 mutants generated, 13 displayed a reduced rate of uptake by HTB94 chondrosarcoma cells. The two mutants (TIMP-3 K26A/K45A and K42A/K110A) with lowest rates of uptake were further evaluated and found to display reduced binding to LRP1 and unaltered inhibitory activity against prototypic metalloproteinases. TIMP-3 K26A/K45A retained higher affinity for sulfated glycosaminoglycans than K42A/K110A and exhibited increased affinity for ADAMTS-5 in the presence of heparin. Both mutants inhibited metalloproteinase-mediated degradation of cartilage at lower concentrations and for longer than wild-type TIMP-3, indicating that their increased half-lives improved their ability to protect cartilage. These mutants may be useful in treating connective tissue diseases associated with increased metalloproteinase activity.

## Introduction

Tissue inhibitor of metalloproteinases-3 (TIMP-3)^2^ is an endogenous inhibitor of metalloproteinase catalytic activity and hence an important regulator of connective tissue turnover by these enzymes (1–3). TIMP-3 is unique among the four mammalian TIMPs in that it can inhibit not only matrix metalloproteinases (MMPs), but also the related adamalysins (a disintegrin and metalloproteinase (ADAMs)) and adamalysins with thrombospondin motifs (ADAMTSs).

Various pathophysiological states are associated with disrupted extracellular matrix (ECM) turnover. For example, osteoarthritis is characterized by degradation of type II collagen and aggrecan by collagenolytic MMPs and aggrecan-degrading ADAMTSs, respectively. *Timp3*-null mice show increased collagenase and aggrecanase activity in cartilage and increased spontaneous osteoarthritis with age (4). TIMP-3 levels are reduced in human osteoarthritic cartilage (5). Treatment with recombinant TIMP-3 has been shown to block cartilage degradation *in vitro* (6) and in *in vivo* models of osteoarthritis (7), further illustrating the chondroprotective activity of TIMP-3.

The half-life of TIMP-3 in tissue is positively regulated by its binding to the ECM (8, 9) and negatively regulated by its endocytosis and subsequent lysosomal degradation via low density lipoprotein receptor-related protein 1 (LRP1) (10, 11). We postulated that a mutant of TIMP-3 with reduced affinity for LRP1 would have a longer half-life in tissue and an increased ability to block metalloproteinase activities compared with wild-type TIMP-3.

Previous mutagenesis (12–14), crystallography (15), and NMR (16) studies on LRP1 ligands have identified a receptor binding motif comprising 2 surface-located lysine residues separated by 21 Å. These lysine residues interact with acidic pockets on two sequential complementary repeats of LRP1 (15). Receptor-associated protein (RAP) is a folding chaperone of the LRP family and has served as a prototypic ligand in many studies investigating LRP-ligand interactions. RAP Lys-256 and Lys-270 are thought to be primarily responsible for binding to LRP1, because the RAP mutant K256A/K270A shows negligible LRP1 binding and endocytosis (13, 14). Other LRP1 ligands, including activated α_2_-macroglobulin (17), factor VIII (18, 19), and the serpins plasminogen activator inhibitor-1 (PAI-1) (20, 21), and nexin-1 (21), have also been shown to utilize lysine residues for LRP1 binding, suggesting that ligands interact with LRP1 though a common mechanism.

To engineer LRP1-resistant mutants of TIMP-3, we analyzed a model of the three-dimensional structure of full-length TIMP-3 and identified pairs of lysine residues potentially separated by 21 Å. These lysine residues were mutated to alanine singly and in pairs, and the endocytosis resistance, LRP1 binding, and chondroprotective ability of the mutants were evaluated. Two of the engineered mutants, TIMP-3 K26A/K45A and K42A/K110A, exhibited substantial endocytosis resistance and protected cartilage better than wild-type TIMP-3. We thus show that targeting the TIMP-3 endocytosis pathway is a potential strategy for inhibiting excess metalloproteinase activity in pathological settings.

## Results

### 

#### 

##### Design of TIMP-3 Mutants

**FIGURE 1.**
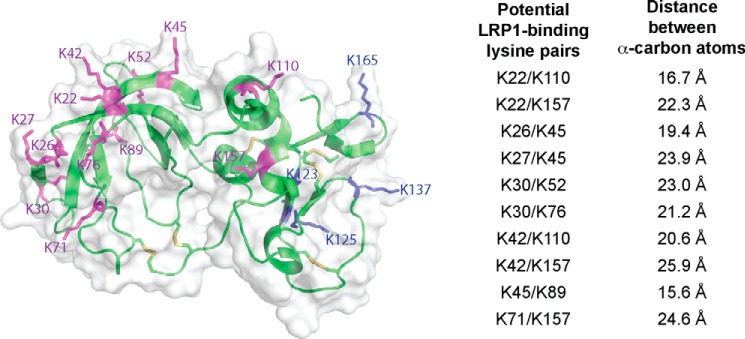
**Identification of potential LRP1-binding residues in TIMP-3.** A model of TIMP-3 was generated using the available crystal structure of TIMP-2. The position of Lys-180 was unresolved in the model, but the remaining 16 lysine residues of TIMP-3 were all predicted to be located on the surface of the molecule. Lysine residues located on the N-terminal domain of TIMP-3 are indicated in *purple*, and those on the C-terminal domain are shown in *blue*. The distance between α-carbon residues of pairs of lysine residues was measured, and 10 pairs of lysine residues (listed on the *right*) were predicted to be separated by 21 ± 5 Å. This figure was made with PyMOL.

Because no crystal structure of full-length TIMP-3 is available, we constructed a homology model of the TIMP-3 structure using the available TIMP-2 (Protein Data Bank code 1BR9) (22) structure. We then compared the N-terminal domain of TIMP-3 in our model with the available crystal structure of the N-terminal domain of TIMP-3 in complex with ADAM17 (Protein Data Bank code 3CKI) (23) and observed good agreement between the two structures. The most C-terminal lysine residue (Lys-180) is unresolved in the model. The remaining 16 lysine residues of TIMP-3 are predicted to be located on the surface of the protein. We measured the distance between α-carbon residues of pairs of lysine residues and identified 10 pairs of lysine residues predicted to be separated by 21 ± 5 Å. (Fig. 1). With the exception of Lys-157, all lysine residues identified were located on the N-terminal inhibitory domain of TIMP-3. Using site-directed mutagenesis, we generated 10 mutants of TIMP-3 in which both lysine residues of the potential LRP1-binding dilysine motif were mutated to alanine as well as 12 mutants in which the individual lysine residues identified were singly mutated to alanine (Table 1). A FLAG tag was included at the C terminus of all mutants for detection and purification, as described previously for wild-type TIMP-3 (24).

**TABLE 1:** **Candidate LRP1-resistant mutants of TIMP-3 generated** Using a model of TIMP-3, 10 pairs of Lys residues were predicted to be separated by 21 ± 5 Å on the surface of TIMP-3, and 10 double Lys → Ala mutants were generated. The 12 constituent single Lys → Ala mutants generated are also listed.

Mutant	Distance between lysine α-carbon atoms
	Å
**Double Lys → Ala mutants of TIMP-3 generated**	
K22A/K110A	16.7
K22A/K157A	22.3
K26A/K45A	19.4
K27A/K45A	23.9
K30A/K52A	23.0
K30A/K76A	21.2
K42A/K110A	20.6
K42A/K157A	25.9
K45A/K89A	15.6
K71A/K157A	24.6

**Single Lys → Ala mutants of TIMP-3 generated**	
K22A	
K26A	
K27A	
K30A	
K42A	
K45A	
K52A	
K71A	
K76A	
K89A	
K110A	
K157A	

##### Screening of Mutants

We carried out an initial screen of the mutants using immunoblotting to evaluate their rate of disappearance from the medium of HTB94 chondrosarcoma cells. These cells secrete a minimal amount of TIMP-3-binding ECM, and disappearance of TIMP-3 from the medium reflects its endocytosis into cells via LRP1 (11).

**FIGURE 2.**
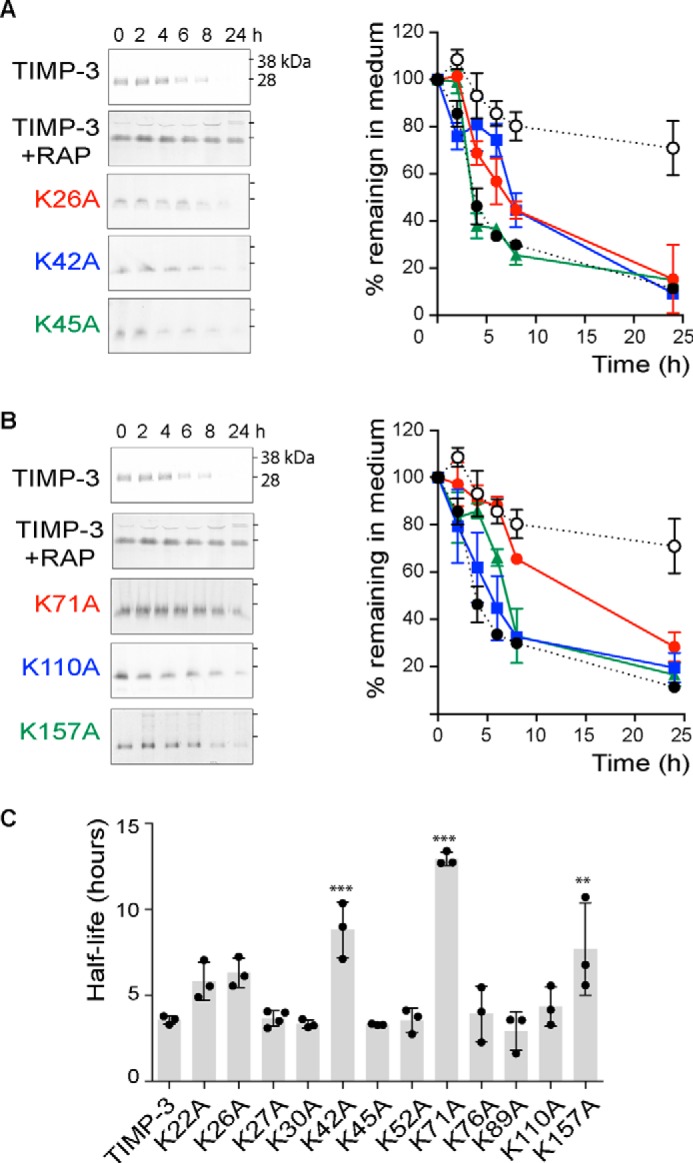
**Mutation of single lysine residues increases half-life of TIMP-3 in the medium of HTB94 chondrosarcoma cells.** *A*, HTB94 chondrosarcoma cells were incubated (0–24 h) with 1 nm FLAG-tagged wild-type TIMP-3 (*black circle*), TIMP-3 preincubated with 1 μM RAP (*white circle*), or TIMP-3 mutant K26A (*red circle*), K42A (*blue square*), or K45A (*green triangle*) in DMEM with 0.1% FCS. Media were concentrated by TCA precipitation and analyzed by immunoblotting with anti-FLAG M2 antibody and densitometry using Phoretix 1D software. The loss of each mutant from the medium was calculated relative to its pixel volume at *t* = 0 h (defined as 100%). Immunoblots show a representative experiment, and graphs show analysis of three technical replicates (mean ± S.D. (*error bars*)). *B*, HTB94 chondrosarcoma cells were incubated (0–24 h) with FLAG-tagged wild-type TIMP-3 (*black circle*), TIMP-3 preincubated with 1 μM RAP (*white circle*), or TIMP-3 mutant K71A (*red circle*), TIMP-3 K110A (*blue square*), or K157A (*green triangle*) in DMEM with 0.1% FCS. Media were concentrated and analyzed as in *A*. Immunoblots show a representative experiment, and graphs show analysis of three technical replicates (mean ± S.D.). *C*, half-lives of wild-type TIMP-3 and its mutants were calculated from *A* and *B.* Wild-type TIMP-3 had a half-life of 3.6 ± 0.3 h. TIMP-3 mutants K42A, K71A, and K157A exhibited significantly increased half-lives of 8.8 ± 1.6, 12.9 ± 0.4, and 7.8 ± 2.5 h, respectively (**, *p* ≤ 0.01; ***, *p* ≤ 0.001).

HEK-293-EBNA cells were transiently transfected with the mutants and conditioned medium dialyzed against TBS. We confirmed that wild-type TIMP-3 in such dialyzed crude conditioned medium was endocytosed by HTB94 with indistinguishable kinetics from purified recombinant TIMP-3, validating this method as a screening tool. Clearance was inhibited by RAP (Fig. 2, *A* and *B*), confirming our previous findings that TIMP-3 is endocytosed in an LRP1-dependent manner (10, 11).

Wild-type TIMP-3 was lost from the medium with a half-life of 3.6 ± 0.3 h (Fig. 2*A*). TIMP-3 K42A, K71A, and K157A exhibited significantly increased half-lives of 8.8 ± 1.6, 12.9 ± 0.4, and 7.8 ± 2.5 h, respectively (Fig. 2*C*). The remainder of the single mutants showed no statistically significant change in half-life, and >75% of all of the single mutants were lost from the medium within 24 h.

**FIGURE 3.**
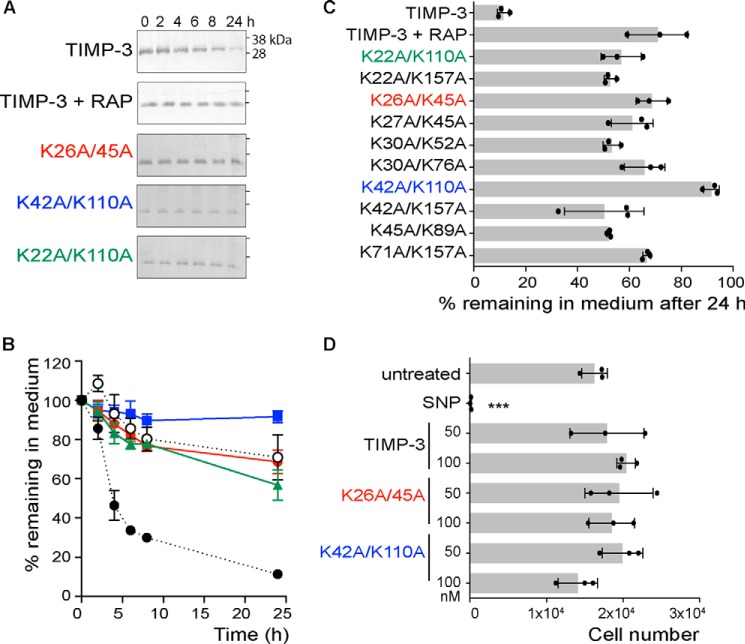
**Mutation of pairs of lysine residues further increases the half-life of TIMP-3 in the medium of HTB94 chondrosarcoma cells.** *A*, HTB94 chondrosarcoma cells were incubated (0–24 h) with 1 nm FLAG-tagged wild-type TIMP-3, TIMP-3 preincubated with 1 μM RAP, TIMP-3 K26A/K45A, TIMP-3 K42A/K110A, or TIMP-3 K22A/K110A in DMEM with 0.1% FCS. Media were concentrated by TCA precipitation and analyzed by immunoblotting with anti-FLAG M2 antibody. The loss of each mutant from the medium was calculated relative to its pixel volume at *t* = 0 h (defined as 100%). *B*, data were analyzed by densitometry using Phoretix 1D software (mean ± S.D. (*error bars*), *n* = 3 technical replicates). Levels of TIMP-3 (*black circle*), TIMP-3 preincubated with 1 μM RAP (*white circle*), and the TIMP-3 mutants K26A/K45A (*red circle*), K42A/K110A (*blue square*), and K22A/K110A (*green triangle*) in the medium are shown. *C*, the percentage of each TIMP-3 double mutant (mean ± S.D., *n* = 3 technical replicates) remaining in the medium at 24 h was calculated. After 24 h, all mutants were present in the medium at significantly (*p* ≤ 0.001) higher levels than wild-type TIMP-3, with K26A/K45A or K42A/K110A remaining in the medium at the highest concentration. *D*, HTB94 chondrosarcoma cells were treated with purified wild-type TIMP-3, K26A/K45A, or K42A/K110A (50 or 100 nm) or sodium nitroprusside (*SNP*; 10 mm) for 24 h, and cell viability (mean ± S.D., *n* = 3 technical replicates) was assessed using the MTS assay. Sodium nitroprusside caused significant cell toxicity (***, *p* < 0.001), whereas wild-type and mutant TIMP-3 had no effect on cell viability at the concentrations tested (*p* > 0.05).

We then analyzed the half-lives of TIMP-3 mutants in which both lysine residues of the potential LRP1-binding dilysine motif were mutated to alanine. All of the double mutants remained in the medium for markedly longer than the single lysine mutants (Fig. 3). More than 50% of each remained in the medium after 24 h, so half-lives could not be calculated, and we compared the mutants based on the percentage remaining in the medium at 24 h. TIMP-3 K42A/K110A and K26A/K45A stayed in the medium at the highest concentration, with 92 ± 3% and 69 ± 6% of these mutants remaining in the medium after 24 h, respectively. These mutants were purified for further characterization.

##### Purification of TIMP-3 K26A/K45A and K42A/K110A

C-terminally FLAG-tagged TIMP-3 mutants were purified by FLAG affinity chromatography as described previously for wild-type TIMP-3 (24). K26A/K45A and K42A/K110A were purified with lower yields (96 and 110 μg per liter, respectively) than wild-type TIMP-3 (288 μg/liter). The remaining 8 TIMP-3 double lysine mutants had yields of <20 μg/liter. We confirmed that K26A/K45A and K42A/K110A retained the same degree of endocytosis resistance as observed previously using crude conditioned media. No cell toxicity was observed after incubation of HTB94 with purified wild-type or mutant TIMP-3 (50 or 100 nm) for 24 h (Fig. 3*D*).

##### Confocal Microscopy Imaging of Endocytosis

**FIGURE 4.**
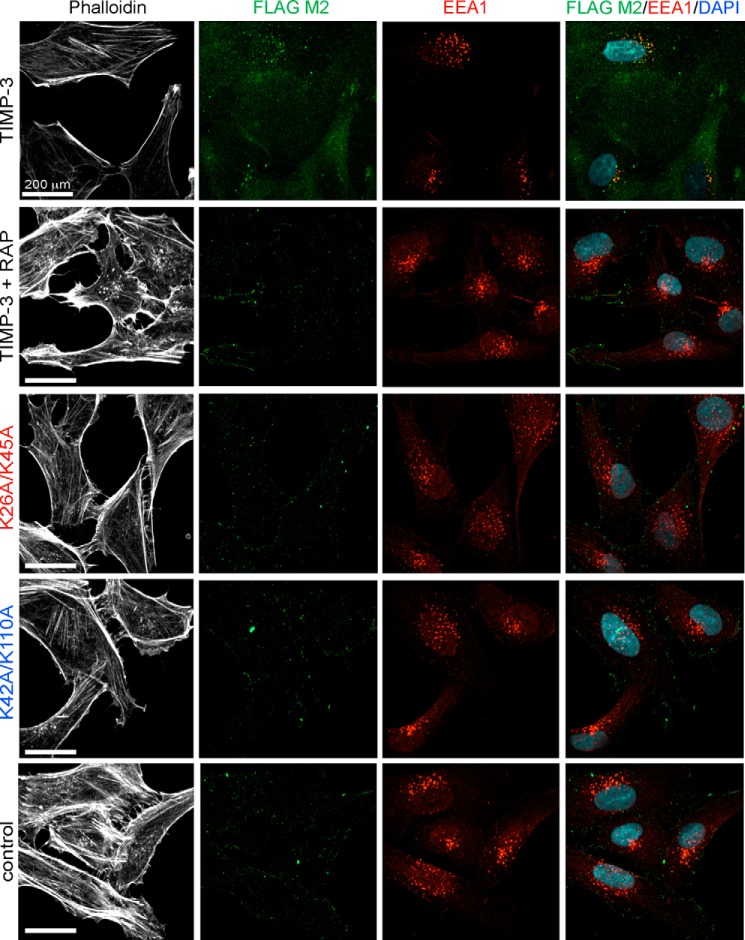
**Wild-type TIMP-3 is endocytosed into lysosomal vesicles, whereas K26A/K45A and K42A/K110A are not.** HTB94 chondrosarcoma cells were grown on gelatin-coated coverslips and incubated (2 h, 37 °C) with TIMP-3, TIMP-3 K26A/K45A, or TIMP-3 K42A/K110A (40 nm) in DMEM with 0.1% FCS. Control wells were incubated without TIMP-3, and LRP dependence was evaluated by incubating wells with RAP (500 nm) for 1 h before the addition of TIMP-3. After fixing and permeabilizing the cells, TIMP-3 was detected with M2 anti-FLAG antibody (*green*), and endocytic vesicles were visualized with an antibody against EEA1 (*red*). Nuclei were visualized by DAPI staining (*blue*), and the cytoskeleton was visualized by staining with phalloidin (*white*). Images were gathered by confocal microscopy on a PerkinElmer Life Sciences spinning disc confocal microscope equipped with a Nikon TE-2000 CCD camera (×40 objective lens).

We added purified wild-type TIMP-3 to HTB94 cells and observed co-localization of the protein with early endosomal antigen 1 (EEA1) in endosomal vesicles (Fig. 4). Preincubation of cells with RAP ablated this co-localization, confirming that TIMP-3 endocytosis is LRP1-dependent. K26A/K45A and K42A/K110A were not visible within cells and did not co-localize with EEA1, confirming that they are not endocytosed.

##### Uptake by LRP1-null Cells

**FIGURE 5.**
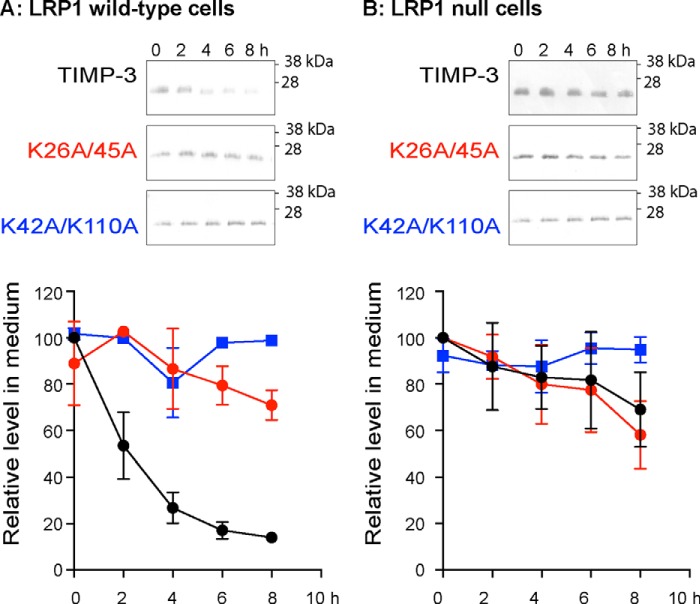
**Uptake of TIMP-3 is LRP1-dependent.** LRP1 wild-type (PEA10; *A*) and LRP1 null (PEA13; *B*) cells were incubated (0–8 h) with FLAG-tagged wild-type TIMP-3, TIMP-3 K26A/K45A, or K42A/K110A (1 nm) in DMEM with 0.1% FCS. Media were concentrated by TCA precipitation and analyzed by immunoblotting with anti-FLAG M2 antibody. The loss of each protein from the medium was calculated relative to its pixel volume at *t* = 0 h (defined as 100%). Immunoblots show representative experiments, and graphs show analysis of three technical replicates (mean ± S.D. (*error bars*)).

We compared uptake of wild-type and mutant TIMP-3 by LRP-1 wild-type and null cells to evaluate whether the altered uptake of the mutants was dependent on LRP1. In LRP1 wild-type PEA10 cells, TIMP-3 was rapidly taken up from the medium with a half-life of 1.9 h, whereas K26A/K45A and K42A/K110A remained in the medium (Fig. 5*A*). Conversely, all three proteins remained at the same level in the medium of LRP1-null PEA13 cells (Fig. 5*B*).

##### Binding to LRP1

**FIGURE 6.**
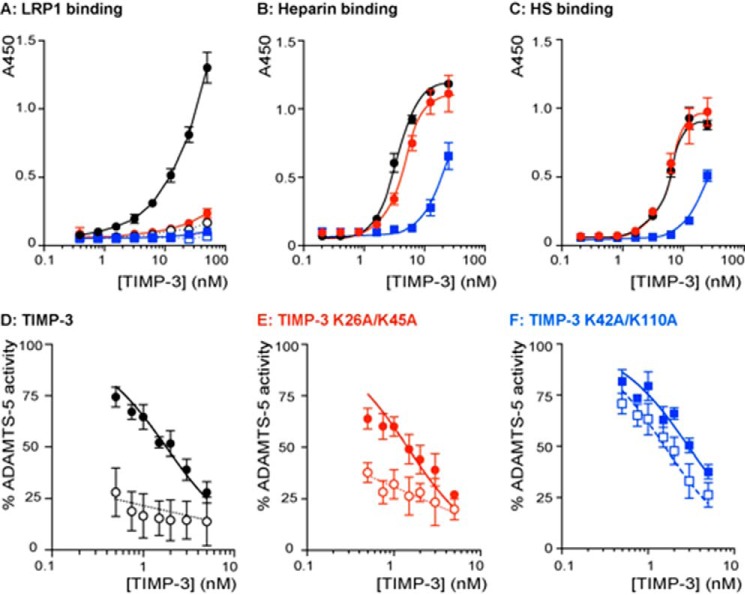
**Characterization of TIMP-3 K26A/K45A and K42A/K110A binding properties.** *A*, binding of wild-type TIMP-3 (*black circle*), K26A/K45A (*red circle*), and K42A/K110A (*blue square*) to LRP1 was analyzed by ELISA. Medium-binding ELISA plates were coated with LRP1 (5 nm) overnight and incubated with 0.4–50 nm wild-type or mutant TIMP-3, either alone (*closed symbols*) or preincubated with heparin (*open symbols*; 150 μg/ml, 1 h, 37 °C). Bound protein was detected using an M2 anti-FLAG primary antibody and anti-mouse-HRP secondary antibody. Hydrolysis of 3,3′,5,5′-tetramethylbenzidine substrate was analyzed by measuring absorbance at 450 nm (mean ± S.D. (*error bars*), *n* = 3 technical replicates). *B*, binding of wild-type TIMP-3 (*black circle*), K26A/K45A (*red circle*), and K42A/K110A (*blue square*) to heparin was analyzed by ELISA. Glycosaminoglycan-binding ELISA plates were coated with heparin (10 μg/ml) overnight and incubated with 0.4–25 nm wild-type or mutant TIMP-3. Bound protein was detected as in *A* (mean ± S.D., *n* = 3 technical replicates). *C*, binding of wild-type TIMP-3 (*black circle*), K26A/K45A (*red circle*), and K42A/K110A (*blue square*) to heparan sulfate was analyzed by ELISA. Glycosaminoglycan-binding ELISA plates were coated with heparan sulfate (10 μg/ml) overnight and incubated with 0.4–25 nm wild-type or mutant TIMP-3. Bound protein was detected as in *A* (mean ± S.D., *n* = 3 technical replicates). *D*, ADAMTS-5 (0.5 nm) was incubated with wild-type TIMP-3 (0.5–10 nm) without (*black circle*) or with heparin (100 nm, *white circle*) for 1 h at 37 °C, and residual enzyme activity was quantified (18 h, 37 °C, mean ± S.D., *n* = 3 technical replicates). *E*, ADAMTS-5 (0.5 nm) was incubated with K26A/K45A (0.5–10 nm) without (*black circle*) or with heparin (100 nm, *white circle*) for 1 h at 37 °C, and residual enzyme activity was quantified (18 h, 37 °C, mean ± S.D., *n* = 3 technical replicates). *F*, ADAMTS-5 (0.5 nm) was incubated with K42A/K110A (0.5–10 nm) without (*blue square*) or with heparin (100 nm, *white square*) for 1 h at 37 °C, and residual enzyme activity was quantified (18 h, 37 °C, mean ± S.D., *n* = 3 technical replicates).

Binding of the mutants to full-length LRP1 was evaluated by ELISA. Wild-type TIMP-3 bound efficiently to LRP1, whereas K26A/K45A bound with markedly reduced affinity, and K42A/K110A displayed minimal binding (Fig. 6*A*).

##### Binding to Sulfated Glycosaminoglycans

Wild-type TIMP-3 had *K_D_* values of 3.7 ± 0.24 and 5.1 ± 0.3 nm for heparin and heparan sulfate, respectively (Fig. 6, *B* and *C*). TIMP-3 K26A/K45A displayed similar affinity, with *K_D_* values of 5.5 ± 0.4 and 5.5 ± 0.5 nm for heparin and heparan sulfate, respectively. TIMP-3 K42A/K110A displayed 5-fold lower affinity, with *K_D_* values of 27.4 ± 5.3 and 25.4 ± 2.8 nm for heparin and heparan sulfate, respectively.

##### Characterization of Mutant Affinity for Prototypic Metalloproteinases

We compared the inhibition kinetics of the K26A/K45A and K42A/K110A mutants with the wild-type inhibitor. As shown in Table 2 (mean ± S.D., *n* = 3–5), *K_i_*(app) values for wild-type TIMP-3 were in agreement with previous findings (24–26), and TIMP-3 K26A/K45A and K42A/K110A both displayed unaltered affinities for the catalytic domains of MMP-1, MMP-2, and MMP-3, as well as ADAMTS-4 lacking the C-terminal spacer domain, ADAMTS-5 lacking the C-terminal thrombospondin domain, and the ectodomain of ADAM17. LRP1 shedding, which is primarily caused by metalloproteinases, was also unaltered by the mutants (not shown).

**TABLE 2:** **Affinity of wild-type and mutant TIMP-3 for prototypic target metalloproteinases** Wild-type TIMP-3 and its mutants K26A/K45A and K42A/K110A (0.25–25 nm) were incubated with target metalloproteinases (1 h, 37 °C), and residual activity against synthetic fluorescent substrates was determined. Equilibrium velocities were used to calculate the apparent inhibition constant *K_i_*(app) (mean ± S.D., *n* = 3–5 technical replicates) using the tight binding equation. ND, too low to be determined.

	*K_i_*(app)		
	Wild-type TIMP-3	TIMP-3 K26A/K45A	TIMP-3 K42A/K110A
	*nm*		
MMP-1ΔC	1.20 ± 0.49	0.52 ± 0.33	0.60 ± 0.30
MMP-2ΔC	0.60 ± 0.32	0.63 ± 0.49	0.60 ± 0.53
MMP-3ΔC	1.20 ± 0.47	0.92 ± 0.19	1.40 ± 0.09
ADAMTS-4	0.19 ± 0.01	0.12 ± 0.04	0.24 ± 0.07
ADAMTS-5	1.27 ± 0.46	0.95 ± 0.55	1.12 ± 0.94
ADAMTS-5 + heparin	ND	ND	1.20 ± 0.42
ADAM17	3.54 ± 1.19	3.78 ± 1.79	2.34 ± 1.40

##### Affinity for ADAMTS-5 in the Presence of Heparin

We have previously shown that sulfated glycosaminoglycans, such as heparin and heparan sulfate, increase affinity between wild-type TIMP-3 and glycosaminoglyan-binding enzymes, such as ADAMTS-5, by mediating formation of high affinity trimolecular complexes (27). As shown in Fig. 6*D* (representative experiment) and Table 2 (mean ± S.D., *n* = 3–5), heparin increased TIMP-3 affinity for ADAMTS-5 from a *K_i_*(app) value of 1.27 ± 0.46 nm to a value too low to be calculated. Heparin similarly increased K26A/K45A affinity for ADAMTS-5 from a *K_i_*(app) value of 0.95 ± 0.55 nm to a value too low to be calculated. In contrast, affinity of K42A/K110A for ADAMTS-5 was not affected by heparin, with *K_i_*(app) values of 1.12 ± 0.94 and 1.20 ± 0.42 nm in the absence and presence of heparin, respectively.

##### Inhibition of Cartilage Degradation

**FIGURE 7.**
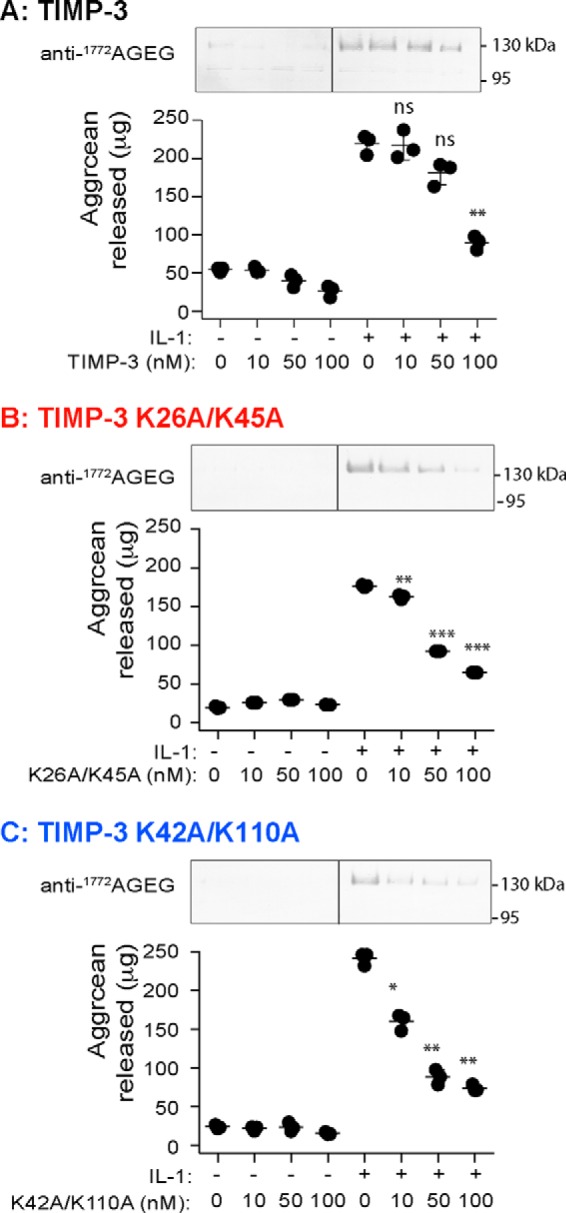
**Effect of TIMP-3 mutants on aggrecan degradation in porcine cartilage explants.** *A–C*, porcine articular cartilage explants were cultured in cartilage medium with 0.1% FCS (250 μl) and/or IL-1 (10 ng/ml) and/or wild-type TIMP-3 (*A*), K26A/K45A (*B*), or K42A/K110A (*C*) (0, 10, 50, or 100 nm). After 2 days, conditioned media were harvested, and aggrecan degradation was analyzed by immunoblotting with the anti-^1772^AGEG neoepitope antibody (representative blot shown) and DMMB assay (graph showing μg of aggrecan released, mean ± S.D. (*error bars*), *n* = 3 biological replicates). *, *p* ≤ 0.05; **, *p* ≤ 0.01; ***, *p* ≤ 0.001 compared with IL-1-stimulated explants. The *vertical line* indicates where a single lane has been cropped from the images.

The ability of the mutants to inhibit IL-1-stimulated aggrecan degradation in porcine cartilage explants was evaluated. In a 48-h assay, glycosaminoglycan release into the conditioned medium was significantly inhibited by wild-type TIMP-3 at 100 nm but not at 10 or 50 nm (Fig. 7*A*). Comparable inhibition was seen using the DMMB dye-binding assay and immunoblotting with the anti-AGEG neoepitope antibody, which detects ADAMTS-mediated cleavage at the TAQE^1771^↓A^1772^GEG site in the aggrecan C-terminal chondroitin sulfate-rich region. K26A/K45A and K42A/K110A inhibited aggrecan degradation at lower concentrations, with significant inhibition observed at 10, 50, and 100 nm concentrations of each mutant (Fig. 7, *B* and *C*). No cell toxicity was observed for any of the treatment conditions (not shown).

**FIGURE 8.**
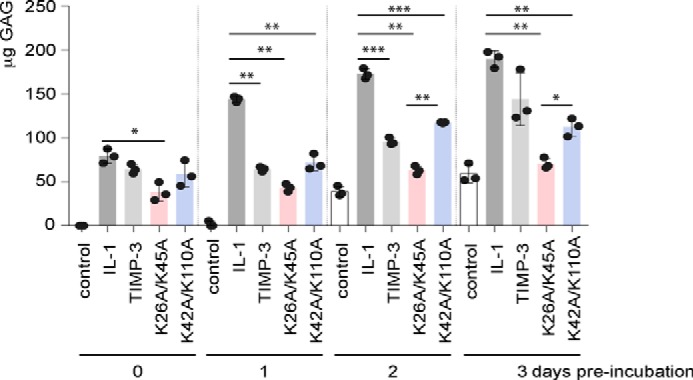
**TIMP-3 K26A/K45A and K42A/K110A protect cartilage for longer than the wild-type inhibitor.** Porcine articular cartilage explants were cultured in cartilage medium with 0.1% FCS (250 μl) and/or wild-type TIMP-3, K26A/K45A, or K42A/K110A (100 nm). After 0, 1, 2, or 3 days, explants were stimulated with IL-1 (10 ng/ml) for 24 h, and aggrecan degradation was analyzed by a DMMB assay (mean ± S.D. (*error bars*), *n* = 3 biological replicates). *, *p* ≤ 0.05; **, *p* ≤ 0.01; ***, *p* ≤ 0.001.

To evaluate the time course of protection, explants were cultured with 100 nm TIMP-3, TIMP-3 K26A/K45A, or TIMP-3 K42A/K110A for 0, 1, 2, or 3 days and then stimulated with IL-1 for a further 24 h. TIMP-3 inhibited aggrecan degradation after 1 and 2 days of preincubation, after which no significant protection was observed (Fig. 8). TIMP-3 K26A/K45A and K42A/K110A, however, significantly protected cartilage against an IL-1 challenge even after 3 days of preincubation on the explants (Fig. 8). K26A/K45A inhibited cartilage degradation significantly more effectively than K42A/K110A after 2 and 3 days of preincubation.

## Discussion

The phenotype of *Timp3*-null mice demonstrates that TIMP-3 is a central inhibitor of metalloproteinase-mediated ECM degradation and that it serves to regulate homeostatic tissue turnover (1–3) and limit pathological tissue remodeling (4). We have previously demonstrated that extracellular levels of TIMP-3 are primarily regulated at the post-translational level, by the balance between its binding to sulfated glycosaminoglycans of the ECM and its cellular uptake by the LRP1 endocytic receptor (28). We sought to engineer a mutant of TIMP-3 with reduced affinity for LRP1 and increased half-life in the tissue, to establish whether tissue remodeling could be modulated by targeting the TIMP-3 endocytic pathway.

Following previous studies that had identified an apparent LRP1-binding motif (13–15), we used a model of the three-dimensional structure of TIMP-3 and identified pairs of lysine residues whose α-carbon atoms are predicted to be separated by 21 ± 5 Å. In agreement with previous findings (13, 14), our initial screening procedure indicated that none of the 12 lysine residues assessed is solely responsible for LRP1 binding. Only three of the single mutants (K42A, K71A, and K157A) displayed significantly longer half-lives. Of these, K71A was the most effective, with a 3.6-fold increased half-life. However, all 10 of the double mutants, in which pairs of lysine residues were mutated to alanine, showed substantially longer half-lives, with 50–90% of the initial concentration remaining in medium after 24 h. The increased half-lives of all double mutants assessed argues against LRP1 binding being governed by a single pair of lysine residues. This is in agreement with the recent findings of van den Biggelaar *et al.* (29), who found that binding of factor VIII to LRP1 was mediated by multiple lysine residues within an extended basic area on the surface of the ligand, leading them to suggest an additive binding model. The crystal structure of RAP in complex with a fragment of the LDL receptor shows that 2 lysine residues of the ligand interact with adjacent acidic pockets on the receptor (15). The extended binding model suggests that this interaction is supported by the surrounding charge landscape and that the interacting lysine residues do not act in isolation. This model may also apply to other LRP1 ligands. For example, double lysine mutants other than RAP K256A/K270A also display reduced affinity for LRP1 (13, 14).

We compared the most endocytosis-resistant of the double mutants, namely K26A/K45A and K45A/K110A, with wild-type TIMP-3 in more detail. Consistent with the lysine residues being distal from the metalloproteinase-binding region of TIMP-3, the mutants showed no change in their ability to inhibit target prototypic metalloproteinases, aggrecanases, and ADAM17. We assessed their biological activity using a cartilage degradation model in which aggrecan degradation is stimulated by treating porcine cartilage explants with the proinflammatory cytokine IL-1. Aggrecan degradation was inhibited by 100 nm wild-type TIMP-3, in agreement with what has been observed previously for the inhibitory N-terminal domain of the inhibitor (6, 30). TIMP-3 K26A/K45A and K45A/K110A inhibited aggrecan degradation more effectively, with significant inhibition observed at lower concentrations and for longer than wild-type TIMP-3. This confirms our hypothesis that LRP1-resistant mutants of TIMP-3 would have an improved ability to inhibit metalloproteinase-mediated extracellular matrix degradation.

TIMP-3 has been reported to induce apoptosis in various cell types (31–33), and we explored the possibility that endocytosis-resistant mutants of TIMP-3 may induce higher levels of apoptosis due to their ability to be retained at higher levels in the extracellular environment. We found that cell viability was not affected by treating chondrosarcoma cells or primary chondrocytes with 100 nm wild-type or mutant TIMP-3 for 2 days, in agreement with previous studies using wild-type N-TIMP-3 (6).

**FIGURE 9.**
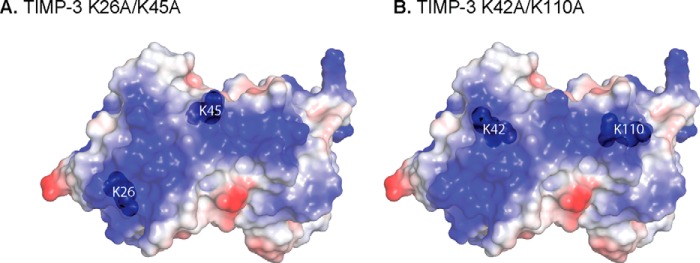
**Model of endocytosis-resistant mutants of TIMP-3.** TIMP-3 contains an extended region of basic residues, depicted as the *blue area* calculated with PyMOL qualitative vacuum electrostatics. This region includes Lys-26/Lys-45 (*A*) and Lys-42/Lys-110 (*B*), whose mutation to alanine reduced TIMP-3 binding to LRP1.

A characteristic feature of TIMP-3 is its ability to bind to sulfated glycosaminoglycans of the extracellular matrix (8, 9). Cell surface HS proteoglycans are also likely to affect the trafficking of TIMP-3 in the extracellular environment. Lee *et al.* (34) demonstrated that this binding was mediated by an extended area of basic residues on the opposite side of TIMP-3 to its inhibitory ridge. The lysine residues in K26A/K45A and K42A/K110A are situated within or adjacent to this basic area (Fig. 9). The mutants retain detectable, albeit reduced, binding to heparin and heparan sulfate, consistent with the requirement to mutate 6 basic residues to ablate ECM binding (34).

Binding of TIMP-3 to sulfated glycosaminoglycans both inhibits its clearance by LRP1 and increases its affinity for glycosaminoglycans-binding target enzymes through formation of high affinity TIMP-3·metalloproteinase·glycosaminoglycan complexes (28). In the context of osteoarthritis, both the collagenase MMP-13 and the aggrecanases ADAMTS-4 and ADAMTS-5 are susceptible to such ECM-mediated increase in TIMP-3 potency (10). Comparing the affinities of the mutants for sulfated glycosaminoglycans and LRP1 gives insight into the relative importance of matrix binding and LRP1-mediated clearance for the biological activity of TIMP-3. TIMP-3 K42A/K110A had greater resistance to endocytosis and lower affinity for LRP1 than was observed for K26A/K45A but also had lower affinity for sulfated glycosaminoglycans and did not display increased affinity for ADAMTS-5 in the presence of heparin. Both mutants protected cartilage more effectively than wild-type TIMP-3, but K26A/K45A showed greater inhibition after extended incubation on cartilage. This suggests that interaction with matrix glycosaminoglyans supports the protective effect of TIMP-3 in the complex, dynamic tissue environment.

Extracellular levels of other LRP1 ligands may be similarly regulated by the equilibrium between matrix binding and LRP1-mediated uptake that we have observed for TIMP-3. For example, heparin inhibits LRP1 binding of factor IXa (35), apolipoprotein A-V (36), C4b-binding protein (37), and PAI-1 (38, 39), and several other LRP1 ligands (*e.g.* ADAMTS-4, ADAMTS-5, MMP-13, TGFβ, CTGF, midkine, thrombospondin) are known to bind to heparin or heparan sulfate (40–46). The bioavailability of these proteins is thus likely to be modulated by factors governing their retention on heparan sulfate proteoglycans, such as the pattern and degree of sulfation on heparan sulfate. Matrix turnover may thus be regulated by the dynamic alterations in sulfation that are observed, for example, with age (47) and inflammation (48).

*Timp3*-null mice also exhibit increased sensitivity to TNF-mediated pathologies, such as impaired liver regeneration (49), indicating the importance of TIMP-3 as a regulator of ADAM17, which sheds membrane-bound TNF to release the soluble form of this pleiotropic proinflammatory cytokine. The effects of K26A/K45A and K42A/K110A on TNF-dependent chronic inflammatory conditions would be of interest. Aggrecanase-selective and ADAM17-selective TIMP-3 mutants with increased half-life could be created by combining the LRP1-resistant mutants described here with previously characterized selective TIMP-3 mutants (30, 50). These mutants or small molecule inhibitors of TIMP-3 endocytosis may be of use in osteoarthritis as well as in other pathological conditions characterized by excess, dysregulated metalloproteinase activity, such as cancer and chronic diabetic wounds.

## Experimental Procedures

### 

#### 

##### Materials

DMEM, penicillin/streptomycin, HEPES, and trypsin-EDTA were from Lonza (Slough, UK). Sodium chlorate, porcine mucosal heparin, amphotericin B, and chondroitin-4-sulfate were from Sigma-Aldrich (Dorset, UK). Hygromycin B was from Calbiochem (Nottingham, UK). Heparan sulfate was from Iduron (Cheshire, UK). FCS was from Gibco (Paisley, UK). Lipofectamine 2000 transfection reagent was from Invitrogen (Paisley, UK).

##### Construction of TIMP-3 Model

ICM-Pro from Molsoft was used to create a homology model of TIMP-3 based on its sequence similarity with TIMP-2 (22). Global pairwise sequence alignment between the homologues was used as the basis for initial structural superposition followed by constrained optimization of side chains and loops. The distance between α-carbon residues of pairs of lysine residues was measured using PyMOL.

##### Generation of TIMP-3 Mutants

TIMP-3 mutants were generated using the QuikChange II XL site-directed mutagenesis kit (Agilent Technologies, Cheshire, UK), with C-terminally FLAG-tagged wild-type TIMP-3 in pCEP4 mammalian expression vector used as the template (24). All mutants generated are shown in Table 1. Primers for generation of K22A were 5′-TCC GAC ATC GTG ATC CGG GCC GCG GTG GTG GGG AAG AAG CTG-3′ (forward) and 3′-AGG CTG TAG CAC TAG GCC CGG CGC CAC CAC CCC TTC TTC GAC-5′ (reverse); primers for K26A were 5′-CGG GCC AAG GTG GTG GGG GCG AAG CTG GTA AAG GAG GGG CCC-3′ (forward) and 3′-GCC CGG TTC CAC CAC CCC CGC TTC GAC CAT TTC CTC CCC GGG-5′ (reverse); primers for K27A were 5′-CGG GCC AAG GTG GTG GGG AAG GCG CTG GTA AAG GAC GGG CCC-3′ (forward) and 3′-GCC CGG TTC CAC CAC CCC TTC CGC GAC CAT TTC CTG CCC GGG-5′ (reverse); primers for K30A were 5′-GTC GTG GGG AAG AAG CTG GTA GCG GAG GGG CCC TTC GGC ACG CTG-3′ (forward) and 3′-CAG CAC CCC TTC TTC GAC CAT CGC CTC CCC GGG AAG CCG TGC GAC-5′ (reverse); primers for K42A were 5′-GGC ACG CTG GTC TAC ACC ATC ACG CAG ATG AAG ATG TAC CAG GC-3′ (forward) and 3′-CCG TGC GAC CAG ATG TGG TAG CGC GTC TAC TTC TAC ATG GCT CCG-5′ (reverse); primers for K45A were 5′-ACG CTG GTC TAC ACC ATC AAG CAG ATG GCG ATG TAC CGA GGC TTC ACC-3′ (forward) and 3′-GAC CAG ATG TGG TAG TTC GTC TGC CGC TAC ATG GCT CCG AAG TGG-5′ (reverse); primers for K52A were 5′-AAG ATG TAC CGA GGC TTC ACC GCG ATG CCC CAT GTG CAG TAC ATC CAC-3′ (forward) and 3′-TTC TAC ATG GCT CCG AAG TGG CGC TAC GGG GTA CAC GTC ATG TAG GTG-5′ (reverse); primers for K71A were 5′-GCT TCC GAG AGT CTC TGT GGC CTT GCG CTG GAG GTC AAC AAG TAC CAG-3′ (forward) and 3′-CGA AGG CTC TCA GAG ACA CCG GAA CGC GAC CTC CAG TTG TTC ATG CTC-5′ (reverse); primers for K76A were 5′-GGC CTT AAG CTG GAG GTC AAC GCG TAC CAG TAC CTG CTG ACA GGT CGC-3′ (forward) and 3′-CCG GAA TTC GAC CTC CAG TTG CGC ATG GTC ATG GAC GAC TGT CCA GCG-5′ (reverse); primers for K89A were 5′-GGT CGC GTC TAT GAT GGC GCG ATG TAC ACG GGG CTG TGC-3′ (forward) and 3′-CCA GCG CAG ATA CTA CCG CGC TAC ATG TGC CCC GAC ACG-5′ (reverse); primers for K110A were 5′-CAG CTC ACC CTC TCC CAG CGC GCG GGG CTG AAC TAT CGG TATC-3′ (forward) and 3′-GTC GAG TGG GAG AGG GTC GCG CGC CCC GAC TTG ATA GCC ATAG-5′ (reverse); and primers for K157A were 5′-GGT TAC CCT GGC TAC CAG TCC GCG CAC TAC GCC TGC ATC CGG-3′ (forward) and 3′-CCA ATG GGA CCG ATG GTC AGG CGC GTG ATG CGG ACG TAG GCC-5′ (reverse). PCR conditions were as per the manufacturer's instructions, except that 100 ng of template was used per reaction, and the extension time per cycle was increased to 150 s/kb (1435 s). PCR products were digested with DpnI and transformed into *E. coli* XL10-Gold ultracompetent bacteria by heat shock, and cells were plated on carbenicillin agar. Candidate colonies were screened by restriction digestion with XhoI and subsequent DNA sequencing (Eurofins MWG Operon, Ebersberg, Germany).

##### Cell Culture

HEK-293-EBNA and HTB94 human chondrosarcoma cells (both from ATCC, Manassas, VA) were maintained in DMEM with 10% FCS, 100 units/ml penicillin, and 100 units/ml streptomycin at 37 °C in 5% CO_2_.

##### Screening of Mutants

For initial screening of the mutants, we evaluated their resistance to clearance from conditioned medium.

HEK-293-EBNA cells were transiently transfected with pCEP4 expression plasmids encoding wild-type or mutant TIMP-3 using Lipofectamine 2000 and incubated in serum-free DMEM with 30 mm NaClO_3_ for 2 days (24). Conditioned media (500 ml) were harvested and dialyzed against TBS (2 × 1 liter, 18 h, 4 °C). The concentration of each mutant was standardized to 1 nm by comparison with purified recombinant TIMP-3 of known, titrated concentration by immunoblotting with an anti-FLAG M2 antibody (F1804-5MG, Sigma-Aldrich, Dorset, UK).

We evaluated clearance of the TIMP-3 mutants from the medium of HTB94 chondrosarcoma cells. HTB94 cells were seeded overnight (8 × 10^5^ cells/well of a 12-well plate) in DMEM with 10% FCS. Wells were washed three times in serum-free DMEM and incubated in DMEM with 0.1% FCS and the dialyzed HEK-293-EBNA conditioned medium containing wild-type or mutant TIMP-3 (1 nm). Samples were harvested at various time points (0–24 h), and proteins were concentrated by the addition of TCA to a final concentration of 5% (18 h, 4 °C) (51) and subsequent centrifugation (13,000 rpm, 10 min, 4 °C). Pellets were resuspended in SDS-PAGE sample buffer and analyzed by immunoblotting with anti-FLAG M2 antibody. Pixel volumes within the linear range of detection were quantified using Phoretix 1D densitometry software (TotalLab, Newcastle-upon-Tyne, UK), and half-life or percentage of wild-type or mutant TIMP-3 remaining in the medium (mean ± S.D.) was calculated relative to their own pixel volume at *t* = 0 h (defined as 100%). Half-lives were calculated by fitting the data to a first order exponential decay model using Prism (GraphPad Software, Inc., La Jolla, CA). Data from three independent experiments were analyzed for statistical significance using one-way ANOVA with Dunnet's post hoc test for multiple comparisons with Prism (GraphPad Software).

Uptake of wild-type and mutant TIMP-3 by LRP1 wild-type (PEA10) and LRP1-null (PEA13) cells was compared. Cells were seeded overnight at 3 × 10^5^ cells/well of a 12-well plate. Wells were washed three times in serum-free DMEM and incubated with purified wild-type or mutant TIMP-3 (1 nm) in DMEM with 0.1% FCS. Conditioned media were analyzed by immunoblotting as described above for HTB94.

##### Purification of Wild-type and Mutant TIMP-3

Wild-type and mutant TIMP-3 were expressed and purified by modification of the protocol described previously (24). HEK-293-EBNA cells were stably transfected with pCEP4 expression plasmids encoding wild-type or mutant TIMP-3 and selected using hygromycin B (100 μg/ml for TIMP-3 mutants, 800 μg/ml for wild-type TIMP-3). Stable cell lines were treated with serum-free DMEM containing 30 mm NaClO_3_ for 48 h. Conditioned medium was harvested, centrifuged to remove cell debris, and passed over an anti-FLAG M2 affinity resin (Sigma-Aldrich) equilibrated in DMEM. The resin was washed in DMEM containing 1 m NaCl and then extensively washed in DMEM. Bound wild-type or mutant TIMP-3 was eluted with FLAG peptide (200 μg/ml) in DMEM and stored at −80 °C in LoBind microcentrifuge tubes (Eppendorf, Stevenage, UK). The purity of isolated TIMPs was confirmed by silver staining (silver staining kit, Pierce), and their active concentrations were determined by titration against a known concentration of MMP-1.

##### Confocal Microscopy Imaging of Endocytosis

HTB94 cells (10^4^ cells) were plated on gelatin-coated coverslips in 12-well plates. Wells were washed in serum-free DMEM and incubated for 2 h at 37 °C with TIMP-3, TIMP-3 K26A/K45A, or TIMP-3 K42A/K110A (40 nm) in DMEM with 0.1% FCS. Control wells were incubated without TIMP-3, and LRP dependence was evaluated by incubating wells with RAP (500 nm) for 1 h before the addition of TIMP-3. Wells were washed three times in PBS after each of the steps hereafter. Cells were fixed with 3% paraformaldehyde plus PBS (10 min, 25 °C), blocked with 5% goat serum plus 3% BSA plus PBS (1 h, 25 °C), and permeabilized in 0.1% Triton X-100 plus PBS (15 min, 25 °C). Cells were stained with mouse anti-FLAG M2 (5 μg/ml F1804-5MG, Sigma-Aldrich) and rabbit anti-EEA1 and 0.5 μg/ml ab2900 (AbCam, Cambridge, UK) antibodies in block (3 h, 25 °C). Bound primary antibodies were detected with anti-mouse DyLight 679 (ThermoFisher, Waltham, MA) and anti-rabbit Alexa Fluor 568 (Molecular Probes, Inc., Eugene, OR) in block (1 h, 25 °C). Nuclei were visualized (1 h, 25 °C) with DAPI (ThermoFisher), and actin was visualized with phalloidin Alexa Fluor 488 (Molecular Probes). Cells were mounted in ProLong Diamond antifade mountant (ThermoFisher) and analyzed on a PerkinElmer Life Sciences spinning disc confocal microscope equipped with a Nikon TE-2000 CCD camera (×40 objective lens).

##### Cell Viability Assays

Cell viability of HTB94 chondrosarcoma cells or porcine cartilage explants was assessed after treatment with wild-type or mutant TIMP-3 (0–100 nm) with or without IL-1 (10 ng/ml) for 24 h in DMEM containing 0.1% FCS. Sodium nitroprusside (10 mm) was used as a control to induce cell death. Media were replaced with fresh DMEM with 0.1% FCS and MTS reagent (3-(4,5-dimethylthiazol-2-yl)-5-(3-carboxymethoxyphenyl)-2-(4-sulfophenyl)-2H-tetrazolium; Promega, Southampton, UK) according to the manufacturer's instructions. Absorbance at 490 nm was measured after 45 min using a FLUOstar OMEGA microplate reader (BMG Labtech, Aylesbury, UK). Data (mean ± S.D., *n* = 3 technical repeats) were analyzed for statistical significance using one-way ANOVA with Dunnet's post hoc test for multiple comparisons.

##### Recombinant Proteins

The catalytic domains of MMP-1 (MMP-1ΔC), MMP-2 (MMP-2ΔC), and MMP-3 (MMP-3ΔC) were prepared as described previously (24, 52, 53). ADAMTS-4 lacking the C-terminal spacer domain and ADAMTS-5 lacking the C-terminal thrombospondin domain were expressed and purified as described previously (54, 55). ADAM17 ectodomain was a gift from Professor Gillian Murphy (University of Cambridge). RAP was prepared as described previously (56).

##### Determination of Apparent Inhibition Constants K_i_(app) of Wild-type and Mutant TIMP-3

The affinity of wild-type and mutant TIMP-3 for prototypic target metalloproteinases was determined under equilibrium conditions, as described previously (27). The assay buffer contained 50 mm Tris·HCl, pH 7.5, 150 mm NaCl, 10 mm CalCl_2_, 0.05% Brij 35, 0.02% NaN_3_, except for ADAM17, which was assayed in 50 mm Tris·HCl, pH 7.5, 25 mm NaCl, 10 mm CalCl_2_, 0.05% Brij 35, 0.02% NaN_3_. Metalloproteinases (0.5–1 nm) were preincubated with wild-type or mutant TIMP-3 (0.25–25 nm) with or without heparin (100 nm) for 1 h at 37 °C, and residual activity was determined (20 min to 18 h) using fluorescence-quenched substrates (from Bachem (Bubendorf, Switzerland) unless otherwise indicated). MMP-1ΔC and MMP-2ΔC were assayed using Mca-Pro-Leu-Gly-Leu-Dap(Dnp)-Ala-Arg-NH_2_ (1.5 μm) (57), MMP-3ΔC using Mca-Arg-Pro-Lys-Pro-Tyr-Ala-Nva-Trp-Met-Lys(Dnp)-NH_2_(1.5 μm) (58), ADAMTS-4 using carboxyfluorescein-Ala-Glu-Leu-Asn-Gly-Arg-Pro-Ile-Ser-Ile-Ala-Lys-*N*,*N*,*N*′,*N*′-tetramethyl-6-carboxyrhodamine (0.5 μm) (59), ADAMTS-5 using *ortho*-aminobenzoyl-Thr-Glu-Ser-Glu-Ser-Arg-Gly-Ala-Ile-Tyr-(*N*-3-[2,4-dinitrophenyl]-l-2,3-diamiopropionyl)-Lys-Lys-NH_2_ (20 μm) (60), and ADAM17 using Abz-Leu-Ala-Gln-Ala-Val-Arg-Ser-Ser-Ser-Arg-Dpa (20 μm; Calbiochem, Watford, UK) (61). End point fluorescence was measured using a Gemini microplate spectrofluorimeter (Molecular Devices, Wokingham, UK) and the apparent inhibition constant *K_i_*(app) (mean ± S.D., *n* = 3 technical replicates) determined from steady-state velocities by fitting the data to the tight binding equation (62) using Prism (GraphPad Software).

##### Binding to LRP1

LRP1 (5 nm; BioMac, Leipzig, Germany) was coated (overnight, 4 °C) onto medium-binding ELISA plates (Greiner Bio-One, Stonehouse, UK) in 20 mm HEPES, 150 mm NaCl, 5 mm CaCl_2_, 0.05% Tween 20, pH 7.4. Wells were blocked with 10% BSA in TNC buffer (50 mm Tris·HCl, pH 7.5, 150 mm NaCl, 10 mm CaCl_2_, 0.05% Brij 35). Wells were washed in TNC buffer containing 0.1% Tween 20 after this and every subsequent step. Purified wild-type or mutant TIMP-3 (0.4–50 nm), either alone or preincubated with heparin (150 μg/ml, 1 h, 37 °C), was applied to wells in TNC buffer containing 5% BSA (3 h, 25 °C). Binding was detected with anti-FLAG M2 primary antibody and anti-mouse-HRP-conjugated secondary antibody in the same buffer. 3,3′,5,5′-tetramethylbenzidine (Becton Dickinson, Swindon, UK) substrate was added, the reaction was stopped when appropriate by the addition of 2 n H_2_SO_4_, and absorbance at 450 nm was measured using a FLUOstar OMEGA microplate reader (BMG Labtech). Data (mean ± S.D., *n* = 3 technical replicates) were analyzed using Prism (GraphPad Software).

##### Binding to Heparin and Heparan Sulfate

Heparin and heparan sulfate (10 μg/ml) were coated (overnight, 25 °C) onto glyosaminoglycan-binding ELISA plates (BD Biosciences, Oxford, UK) in PBS, and wells were blocked with 0.2% gelatin in PBS (1 h, 37 °C). Wells were washed in PBS containing 0.1% Tween 20 after this and every subsequent step. Purified wild-type or mutant TIMP-3 (0.2–25 nm) in blocking solution was applied to wells (3 h, 37 °C), and binding was detected with anti-FLAG M2 primary antibody and anti-mouse-HRP-conjugated secondary antibody. 3,3′,5,5′-Tetramethylbenzidine (Becton Dickinson) substrate was added, the reaction was stopped when appropriate by the addition of 2 n H_2_SO_4_, and absorbance at 450 nm was measured using a FLUOstar OMEGA microplate reader (BMG Labtech). Data (mean ± S.D., *n* = 3 technical replicates) were analyzed using Prism (GraphPad Software).

##### Cartilage Explant Cultures

Porcine articular cartilage explants were dissected from metacarpophalangeal joints of 3–9-month-old pigs within 24 h of slaughter using a biopsy punch to ensure uniformity of size. Explants were rested for 2 days in cartilage medium (DMEM containing 100 units/ml penicillin, 100 units/ml streptomycin, 2 mg/ml amphotericin B, 10 mm HEPES) with 10% FCS (500 μl of medium, 48-well plates) at 37 °C in 5% CO_2_. Cartilage explants were then washed into cartilage medium (250 μl) with 0.1% FCS and treated with IL-1 (10 ng/ml, PeproTech, London, UK) and/or wild-type or mutant TIMP-3 (0–100 nm) for 0–72 h. IL-1 treatment stimulated catabolic pathways leading to degradation of the cartilage ECM by TIMP-3 target enzymes and release of degraded aggrecan fragments from the cartilage into the conditioned medium (63). Conditioned media were harvested, and aggrecan degradation was quantified (3 technical replicates per condition) using the dimethylmethylene blue (DMMB) dye-binding assay that quantifies chondroitin sulfate-rich fragments of aggrecan released into the medium though the action of ADAMTSs (64). Data from experiments with three biological replicates were analyzed for significance using one-way ANOVA with Dunnet's post hoc test for multiple comparisons.

Aggrecan fragments released into the medium were also analyzed by immunoblotting (2 technical replicates per condition) using a neoepitope antibody that recognizes the new N terminus generated by ADAMTS-dependent cleavage of aggrecan at the TAQE^1771^↓A^1772^GEG site (10).

## Author Contributions

L. T. and H. N. conceived the study, coordinated the work, and wrote the paper. D. D. generated the TIMP-3 model, R. V. designed the mutants, D. K. S. provided the LRP1-null cells, and C. M. D. generated and analyzed the mutants.

## Footnotes

2 The abbreviations used are:

TIMP: tissue inhibitor of metalloproteinases
ADAM: a disintegrin and metalloproteinase
ADAMTS: adamalysin with thrombospondin motifs
DMMB: dimethylmethylene blue
ECM: extracellular matrix
LRP: lipoprotein receptor-related protein
MMP: matrix metalloproteinase
RAP: receptor-associated protein
ANOVA: analysis of variance
Mca: (7-methoxycoumarin-4-yl) acetic acid
Nva: norvaline
Dnp: 2,4-dinitrophenyl
Abz: 2-aminobenzoic acid
Dap: L-2,3-diaminopropionic acid
Dpa: N-3-(2,4-dinitrophenyl)-L-α,β-diaminopropionyl.
